# Supplementary material for: Acceptance and compliance with micronutrient powder (MNP) among children aged 6–23 months in northern Nigeria
Source: PLOS Glob Public Health. 2022 Oct 17;2(10):e0000961. doi: 10.1371/journal.pgph.0000961 (PMC10022258; doi:10.1371/journal.pgph.0000961)
Supplement: S1 Dataset — (ZIP) [file pgph.0000961.s008.zip › S8_Dataset/Spot Checks.zip!/Spot Checks/110715_K_DW_TA_2_SC.pdf]

SPOT CHECK RECORDING FORM

State: KEBBI LGA: DANKO/WASAGU Community: HAYIN HARI

Sex of index child: M   Age of index child (in months): 12

Spot Check Information

1. **Date of MNP distribution to household** (circle option below based on LGA name above)**:**
   - **Kebbi:** BirninKebbi&Jega LGA: **June 16**
   - **Kebbi:** Danko-wasagu: **June 17**
   - **Adamawa:**Song, Ganye: **June 18**
   - **Adamawa:**Guyuk **June 19**
2. **Date of Spot Check Observation: 11TH JULY, 2015_**
3. **Number of sachets remaining**(Ask the caregiver to show you and count the specific number of sachets remaining)**:**

   37

4. **Number of sachets that should remainusing one MNP sachet per day** (Analyst only)**:**

   37

5. **Number of sachets remaining / number of sachets should be remaining**(Analyst only)**:**

____37_____ / ___37______ = _____100____ %

Probes

Question 1. You have **XX** MNP sachets remaining. Explain how easy or difficult it was to use the MNP product on a daily basis for your child 6 – 23 months? (Circle one answer)

Very Difficult **Difficult** **Easy** **Very Easy**

Question 2. Describe any specific challenges that you faced that made the MNP difficult to use on a daily basis?

There was no challenge at all.

Question 3. Describe specific aspects of the MNP product that you or your child enjoyed that made it easier to use on a daily basis?

Because it has been keeping my child healthy, not until today that I observed that he is ill. It has been working well for him and that is what we enjoy.

Question 4. What questions do you have about the MNP based on your experience using it?
I have no question at the moment.

Question 5. What specific recommendations do you have that we can do as a program to make the MNP easier to use on a daily basis?

My recommendation is that there should be medicine that my children will take.
